# Should Health Organizations Use Web 2.0 Media in Times of an Infectious Disease Crisis? An In-depth Qualitative Study of Citizens’ Information Behavior During an EHEC Outbreak

**DOI:** 10.2196/jmir.2123

**Published:** 2012-12-20

**Authors:** Lex van Velsen, Julia E.W.C van Gemert-Pijnen, Desirée J.M.A Beaujean, Jobke Wentzel, Jim E van Steenbergen

**Affiliations:** ^1^National Coordination Centre for Outbreak ManagementNational Institute for Public Health and the EnvironmentBilthovenNetherlands; ^2^Center for eHealth Research and Disease ManagementDepartment of Psychology, Health and TechnologyUniversity of TwenteEnschedeNetherlands; ^3^Centre for Infectious DiseasesLeiden University Medical CentreLeidenNetherlands

**Keywords:** Disease Outbreaks, Foodborne Diseases, Health Communication, Information Dissemination, Information Seeking Behavior, Social networking

## Abstract

**Background:**

Web 2.0 media (eg, Facebook, Wikipedia) are considered very valuable for communicating with citizens in times of crisis. However, in the case of infectious disease outbreaks, their value has not been determined empirically. In order to be able to take full advantage of Web 2.0 media in such a situation, the link between these media, citizens’ information behavior, and citizens’ information needs has to be investigated.

**Objective:**

The goal of our study was to assess citizens’ Web 2.0 media use during an infectious disease outbreak and to determine which Web 2.0 medium is used for which goal. With this information, we wanted to formulate recommendations for health organizations that consider using Web 2.0 media as part of their communication strategy during an infectious disease outbreak.

**Methods:**

A total of 18 student participants kept an information diary for 4 weeks during the 2011 enterohemorrhagic E. coli (EHEC) outbreak in Germany. Of them, 9 lived at the epicenter of the outbreak and 9 of them at some distance. The diaries were supplemented by a qualitative pre-survey (demographics) and postsurvey (questioning their satisfaction with information provision during the outbreak).

**Results:**

The Internet appeared to be the most popular medium for passively receiving EHEC-related information, with news websites and websites of newspapers as the most consulted sources. Twitter was used for receiving information to a small degree, while Facebook played virtually no role. Participants indicated that they thought information posted on Twitter or Facebook was not reliable or was out of place. When actively seeking information, online newspapers and wikis were important sources. Several causes for (dis)satisfaction with information provision were uncovered: source credibility, contradicting messages, and a need for closure.

**Conclusions:**

During an infectious disease outbreak, our small sample of students did not see social media (like Facebook and Twitter) as suitable or reliable sources for communicating information, but primarily viewed them as a tool for communicating with friends. Wikis, however, did fill several information needs, especially when citizens are actively searching for information. For many, source credibility is an important asset of information usefulness. Finally, we provide several general recommendations for communicating with citizens during an infectious disease outbreak.

## Introduction

Crisis situations drastically alter the context in which public health organizations communicate with citizens. The course of events in these situations is highly unpredictable, the stakes are high, citizens are aroused or stressed, and the media is eager for breaking news [1]. Public health organizations need to keep the public informed about the situation in general and should instruct individuals on how to act in times of a health crisis [2]. In the case of an infectious disease outbreak, it also of vital importance that citizens are persuaded to comply with health advice in order to minimize the spread of the infection. Following the definition of the World Health Organization, we see an infectious disease outbreak as a situation in which the occurrence of cases of disease is in excess of what would normally be expected in a defined community, geographical area, or season [3]. Research on risk communication has provided public health officials and communicators with a large set of guidelines for achieving these goals. For example, in the initial phase of an outbreak, one should inform the public about the risks involved in the simplest terms, while citizens should be taught to understand the risks they run themselves in the following, so-called, “crisis maintenance” phase [4].

One crucial aspect of communication during an infectious disease outbreak is selecting the communication channels that will have the highest degree of coverage and impact among the target populations and to tailor messages towards their context. By means of a large-scale telephone survey, Avery [5] uncovered that for American citizens, physicians are the preferred source of information due to their expertise and credibility, followed by television news broadcasts. During the A(H1N1) influenza outbreak in 2009, the three main sources of information for Malaysian citizens appeared to be the newspaper, television, and family members. Their main information needs were information on how to prevent and treat an infection [6]. In the Netherlands, this outbreak taught us that, in order to increase compliance with preventive measures and to gain trust, health organizations should constantly keep the public updated, especially about things that are uncertain [7]. The 2003 SARS outbreak showed that the Dutch obtained information mostly from television and newspapers [8], while a study in Finland indicated that very active media coverage triggers citizens’ interest and increases their knowledge [9].

The rise of Web 2.0 media (such as Facebook, Twitter, and Wikipedia) has offered new possibilities for communicating with and learning from citizens during an infectious disease outbreak. The latter, infodemiology, deals with automatically analyzing user behavior (eg, search behavior) or user-generated content (eg, tweets) in order to detect outbreaks and to inform health professionals [10]. Although the interest of researchers in infodemiology has exploded in recent years [11,12], studies uncovering citizens’ use of 2.0 media in order to fulfill their information needs during an infectious disease outbreak are lacking. However, social media did appear to be valuable in different crisis situations like disaster relief [13] and the uprisings across the Middle East and North Africa [14].

This study focuses on uncovering citizens’ information behavior in times of an infectious disease outbreak, with a special interest in the use of Web 2.0 technologies. We see information behavior as:

The totality of human behavior in relation to sources and channels of information, including both active and passive information seeking, and information use. Thus, it includes face-to-face communication with others, as well as the passive reception of information as in, for example, watching TV advertisements, without any intention to act on the information given. [15]

This definition implies that information behavior is a very broad concept and includes a person’s rationale for using a specific communication channel or source, his or her usage of information and information search technologies, and a person’s evaluation of information. By applying this broad interpretation, we will able to understand exactly why and how a person makes use of information during an infectious disease outbreak.

At the time of our research, this topic of investigation had received no scholarly attention. Thus, we conducted a novel and explorative study and asked 18 persons to keep a diary during a large international EHEC outbreak in which they described what information they received about EHEC, with whom they talked about EHEC, and how they searched for an answer on questions they had about EHEC. The results we gathered allowed us to formulate recommendations for health communicators who are dealing with an infectious disease outbreak and who have to create a communication strategy in which they aim to use Web 2.0 technologies to their maximum potential. Our research question was: Do citizens use 2.0 media during an infectious disease outbreak for being kept up to date and to find answers to their questions, and if so, which 2.0 medium is used for which goal?

### Case: the EHEC-Outbreak

The enterohemorrhagic E. coli (EHEC) bacterium is transmitted via the consumption of contaminated foods or by direct contact with patients. It can cause abdominal cramps, (bloody) diarrhea, fever, and vomiting. For most patients it is a self-limiting disease, but it is serious in some patients (mostly the elderly and young children). Infection can lead to the hemolytic-uremic syndrome (HUS) in 20% of reported cases. HUS shows acute kidney failure and is lethal in 3-5% of diagnosed cases. Worldwide, several large EHEC outbreaks have been reported, for example, the Japanese outbreak in 1996 with 6561 schoolchildren infected after eating lunch prepared with contaminated white radish sprouts [16].

In May 2011, a large EHEC outbreak started in Germany. By the end, 3816 patients were diagnosed with EHEC, of which 845 were with HUS; 40 patients died as a result of the infection [17]. The peak of the outbreak was on 21^st^and 22^nd^of May 2011, and the outbreak ended in June 2011. The epicenter of the outbreak was in the northeastern region of Hamburg. Related cases were also diagnosed in France, Sweden, the United States of America, and the Netherlands [18]. In the Netherlands, bordering the epicenter region, only a few cases linked to the outbreak were registered (11 EHEC cases, of which 4 were HUS cases, and no deaths) [19]. In the end, the source of the outbreak turned out to be fenugreek sprouts [20] from a contaminated batch originating in 2009 from Egypt [21].

Media coverage of the EHEC outbreak was extensive. The vehicle of transmission was unknown for a long time, and speculations dominated the news reports. Furthermore, official public persons and organizations in Germany fed this speculation by giving warnings about the safety of certain types of food (eg, cucumbers, tomatoes, and lettuce), which they later had to retract as that turned out to be untrue. In the Netherlands, at a distance from the epicenter and with only low numbers of patients, it sufficed for the Public Health Institute to provide regular updates with the number of reported cases and with information that there was no risk involved in consuming any type of Dutch food.

An infectious diseases outbreak usually begins as a local incident. In the Netherlands, during a local outbreak, the task of communicating lies initially with the local health authorities. Based on the information at the Municipal Health Service, the Ministry of Health, Welfare and Sport needs to be informed. The Ministry decides, in consultation, when it is necessary to “scale” communication to the national level. When there is an imminent outbreak of an infectious disease, professionals communicate about the threat and the policies to prevent the outbreak. Their communication message focuses on the precautions taken and the resources the government has deployed to monitor the outbreak. Once the outbreak is a “fact”, the emphasis shifts to communicating about the outbreak itself: how can a citizen recognize disease patterns and what can he or she do to prevent further spread of the disease. In Germany, local health offices send reports about (imminent) outbreaks to state ministries. These ministries then send the information to the Robert Koch Institute (RKI), which takes on the involved laboratory investigations. The German Federal Institute for Risk Assessment performs the outbreak investigation. The two latter parties both communicate with the public about their findings.

## Method

### Diary Study

In order to assess EHEC-related information behavior during the 2011 outbreak, we conducted a diary study with a running time of 4 weeks. The collection of diaries allowed us to gain in-depth insight into the information behavior of our participants and to determine whether one event could lead to another (eg, the television news leaving a person with a question who then looks for an answer on Wikipedia) [22]. We used two kinds of diary methods, each with a specific diary entry form (classification by Wheeler and Reis [23]):

1. An interval-contingent form. We asked participants to report their passive consumption of EHEC-related information (eg, seeing a report about the EHEC outbreak on the evening news). Furthermore, they had to report their conversations with other people about EHEC. This had to be done on a daily basis.
2. An event-contingent form. We asked participants to report their active information behavior when a question about EHEC arose and they actively searched for information.

The interval-contingent diary form can be found in Multimedia Appendix 1. It allowed the participants to describe any EHEC-related information they consumed via TV, radio, newspapers, the Internet, and elsewhere. There was also room to describe who they talked to about EHEC and what this conversation was about. The event-contingent diary was based on a diary form by Price and colleagues [24] and can be found in Multimedia Appendix 2. On this form, participants were requested to enter when the search took place, what question triggered it and how important it was for them, where they found an answer, and how satisfied they were with it. Both forms were converted into eForms.

Before participants could take part in the study, we asked them to complete a demographics questionnaire and an informed consent form. They were also ensured of their anonymity in this study. As diary study participants often find it difficult to expect what will be requested of them [22], we sent along an instruction booklet with the diary forms, including examples of completed forms. Next, we asked the respondents to update their interval-contingent diary form at the end of each day. They needed to complete an event-contingent form every time they conducted a search on EHEC-related information. Every week, we asked them to email us the forms with their entries, after which they started on a new form. After they submitted their final diary forms, they were asked to complete a questionnaire about their satisfaction with the information provision about EHEC, their preferred source for EHEC-related information in the previous 4 weeks, and their reasons for (not) using social media in order to receive EHEC-related information. The study lasted from June 10 to July 7, 2011.

### Recruitment of Participants

Participants were recruited from two student populations: one from Hamburg (the outbreak epicenter in Germany), and one from Twente (a region in the Netherlands bordering the Hamburg region). We selected these regions to map the information behavior of those directly affected by the outbreak and people indirectly affected by it; Vartti et al [9] have shown that the proximity of the outbreak affects media coverage and citizen interest. Students were selected as participants for two reasons. First, they were “heavy users” of Web 2.0 technology and thus, could provide us with a thorough understanding of the potential of these technologies in times of an infectious disease outbreak. Second, it was extremely difficult to find participants during the outbreak period to take part in a longitudinal diary study. Recruitment of students using a financial incentive worked well in the required short time period. German participants were recruited via a convenience sample and Dutch participants via a study participant pool.

Initially, we recruited 20 participants (10 in Hamburg and 10 in Twente). After the completion of the demographics questionnaire, 2 participants (1 in Hamburg and 1 in Twente) decided not take part in the study. As the requested time and effort were large, participants received a payment of €50 on submission of a complete diary.

### Analyses

All participants’ entries on their diary forms and questionnaires were recorded in a Microsoft Excel database. The classification of closed questions (eg, senders of passively consumed information such as radio channels) was done deductively by two authors (LvV & JW), and any conflicts were resolved by means of a discussion. The analysis of open-ended questions on the diary forms was done via thematic analysis. If the body of data was small and coding reliability could not be assessed, data were interpreted by two authors (LvV & JW). Themes were assessed deductively, following guidelines by Braun and Clarke [25]. In order to code the content of each message that was consumed by the participants (a large body of data), a coding scheme was created deductively. Following guidelines by Pope, Ziebland, and Mays [26], we took the following steps:

1. One author (LvV) familiarized himself with the data and created a first coding scheme.
2. One author (LvV) then coded all messages using this coding scheme. Whenever a category needed to be altered or a new category needed to be added, he redid the coding of data from the start.
3. When all data were coded without needing to alter the coding scheme, one author (JW) coded a subset of the data (50 entries) with the coding schemes.
4. Disagreements were discussed, which led to alterations to the coding scheme. This coding scheme was finalized and can be found in Multimedia Appendix 3.
5. One author (LvV) recoded all entries using this final coding scheme and a second author (JW) independently recoded 100 entries. On this basis, Cohen’s kappa was calculated at .73. According to Landis and Koch [27], this stands for substantial to almost perfect agreement.

## Results

### Demographics

The participants were studying a variety of subjects, including communication sciences, psychology, and mechanical engineering. Six of the German participants were studying health sciences. Table 1 displays the participants’ demographics. It shows that about half had a newspaper subscription, which is in line with the Dutch [28] and the German [29] average. Television consumption was slightly below average for the Dutch participants [30], as well as for the German participants [31]. Radio consumption was slightly above average for the Dutch participants [30], but below average for the German participants [32]. Finally, Internet use was far above average for the Dutch participants [30] and above average for the German participants [32]. The use of Web 2.0 services, such as Facebook, Twitter, Hyves (a Dutch social network), and StudiVZ (a German social network), among both Dutch and German participants was very high, which is normal for the age group of our respondents [33,34]. As Table 2 shows, there is a high variation in Twitter use among our participants. Two German participants already subscribed to tweets from @EHEC_Watch, a feed about EHEC from the Helmholtz Centre for Infection Research. The other participants did not subscribe to Twitter feeds on health information.

**Table 1:** Participant demographics.

	Sex	Age	Newspaper subscription	Television consumption (hrs/day)	Radio consumption (hrs/day)	Internet use (hrs/day)	Which 2.0 media do you visit?	Which 2.0 media do you have an account with?
NL1^a^	M	19	Yes	0–1	1–4	1–4	Facebook / Twitter	Facebook / Twitter
NL2	F	22	No	2–4	4–8	1–4	Hyves / Facebook / Twitter	Hyves / Facebook / Twitter
NL3	F	21	Yes	0–1	0–1	4–8	Facebook / Twitter	Facebook / Twitter
NL4	F	19	No	0–1	4–8	4–8	Hyves / Facebook / Twitter	Hyves / Facebook / Twitter
NL5	F	21	No	1–2	4–8	4–8	Hyves / Twitter	Twitter
NL6	F	22	No	1–2	1–4	4–8	Hyves / Facebook / Twitter	Hyves / Facebook / Twitter
NL7	M	22	Yes	Never	1–4	4–8	Facebook / Twitter	Facebook / Twitter
NL8	F	22	Yes	Never	4–8	> 8	Facebook / Twitter	Facebook / Twitter
NL10	F	26	No	2–4	1–4	1–4	Twitter	Twitter
G1	F	25	No	2–4	0–1	1–4	Facebook / Twitter	Facebook / Twitter
G2	F	27	Yes	1–2	0–1	1–4	StudiVZ / Facebook / Twitter	StudiVZ / Facebook / Twitter
G4	F	21	No	1–2	0–1	4–8	StudiVZ / Facebook / Twitter	StudiVZ / Facebook / Twitter
G5	F	32	No	0–1	0–1	1–4	Facebook / Twitter	Facebook / Twitter
G6	F	28	Yes	0–1	0–1	1–4	Facebook	Facebook / Twitter
G7	M	22	Yes	2–4	0–1	1–4	Facebook / Twitter	Facebook / Twitter
G8	F	20	No	1–2	1–4	1–4	StudiVZ / Facebook / Twitter	StudiVZ / Facebook / Twitter
G9	F	28	No	1–2	0–1	1–4	Facebook	Facebook / Twitter
G10	F	24	Yes	1–2	0–1	1–4	StudiVZ / Facebook	StudiVZ / Facebook / Twitter

^a^NL = Dutch participants; G=German participants.

**Table 2:** Participants’ Twitter activity (data gathered June 20, 2011).

	Tweets	Number of Twitter feeds followed	Number of followers	Health/EHEC related streams followed^a^
NL1	9,084	418	237	none
NL2	11	22	16	none
NL3	0	17	5	none
NL4	1,870	111	110	none
NL5	1	10	2	none
NL6	3	12	11	none
NL7	[protected]	7	1	[protected]
NL8	28	54	48	none
NL10	909	199	172	none
G1	1	17	4	none
G2	0	17	6	none
G4	0	7	2	none
G5	6	4	2	none
G6	4	3	3	none
G7	[protected]	9	1	[protected]
G8	4	9	4	@EHEC_Watch
G9	0	5	2	@EHEC_Watch
G10^b^	50	44	15	none

^a^Search among people followed on Health/EHEC/gezond/gesund.^b^Participant used someone else’s account on Twitter; data are for holder of Twitter account.

At the start of the diary study, all of the participants had heard of the EHEC bacteria, but they found it difficult to remember where and when they first heard of it. Four German participants recalled first hearing of EHEC through the mass media (radio or TV) in May. Only one Dutch participant could answer this question (he first heard of it on June 1^st^, having read about it in the newspaper).

### Passive Reception of Information

Passive reception of information deals with information that was passively consumed by the participants: no active searches preceded the consumption of information. Most reports of passive reception were made by the German participants at 146. The Dutch participants reported 93 instances of information consumption.

**Figure 1:**
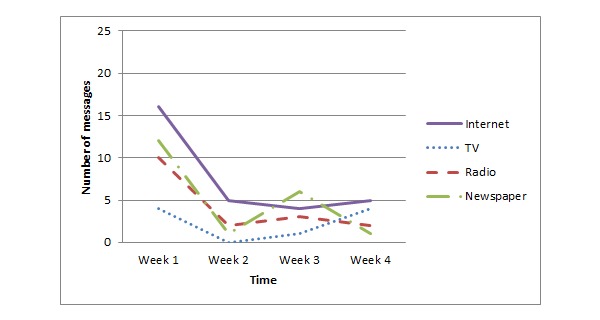
EHEC-related Information consumption by Dutch participants.

**Figure 2:**
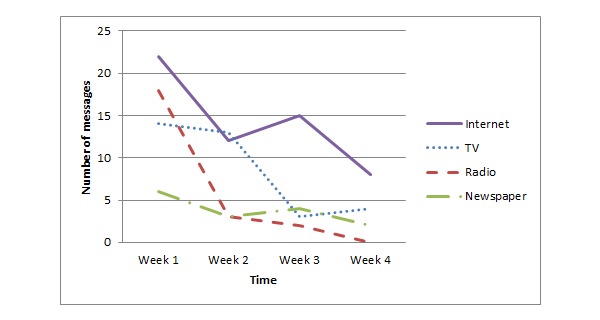
EHEC-related Information consumption by German participants.

Figure 1 shows that Dutch participants mainly consumed EHEC-related information provided via the Internet, followed by information provided by radio and newspapers. During the 4 weeks of data collection, the number of messages consumed by the participants decreased, in line with the decreasing number of messages provided by the media as the outbreak decreased. A somewhat different picture emerged from the German respondents (see Figure 2). In line with the Dutch participants, the Internet was the source that provided most EHEC-related information. However, the German participants also received a lot of information via the TV. Radio and newspapers were least popular.

Next, we took a closer look at the actual online sources of information where the participants passively consumed EHEC-related information. As Table 3 shows, most information was stumbled upon on a news website. In particular, one popular Dutch news website (nu.nl) accounted for 23% of information passively consumed via the Internet. Interestingly, websites hosted by traditional media outlets were also very popular: websites of newspapers or newsmagazines accounted for 30% of the total passively consumed online information, followed by websites of TV channels or networks (16%). Facebook was a social medium that delivered virtually no information on EHEC to the participants (1%). Twitter, on the other hand, delivered 10% of the passively consumed online information. It needs to be said that 7 out of these 10 instances were Tweets sent by either a Twitter feed managed by a newspaper or a TV channel (for the other 3, the source could not be established). None of these tweets were sent by @EHEC_Watch.

**Table 3:** Internet sources that provided information passively (n=100).

Source	Percentage of total	Number of unique websites
News website	35%	8
Website newspaper / newsmagazine	30%	17
Website TV channel / TV network	16%	6
Website academic journal	3%	2
News website with discussion forum	2%	1
(Semi) Government website	2%	2
Website health insurance company	1%	1
Twitter	10%	
Facebook	1%	

After looking at the media and sources of passively consumed information, we analyzed the content of these messages. The Dutch participants reported 129 messages, the German participants 235 messages. Tables 4 and 5 display the message subjects that were transmitted most, divided over the media that facilitated the transmissions. The following themes were reported most (terms in brackets refer to column headings in Tables 4 and 5):

1. Presence EHEC bacteria (Presence). Messages about the (proven or not proven) prevalence of the EHEC bacteria in a country, on a product (group), on a company, or in a river, or messages about the starting point of the EHEC outbreak
2. Preventive measures (Preventive). Messages about preventive measures that are being taken to prevent the spread of, or infection with the EHEC bacteria, including messages about the (un)safety of a specific product group
3. Number of deaths (Deaths). Messages about the number of deaths as a result of the EHEC outbreak
4. Number of infections (Infections). Messages about the number of infections as a result of the EHEC outbreak
5. Geographical spread (Spread). Messages about a specific area where the EHEC bacteria has been encountered or could be present for the first time, where people have become ill for the first time, or have died for the first time as a result of the EHEC bacteria
6. Pathogenesis of EHEC (Pathogenesis). Messages about the way the EHEC bacteria spreads or infects a human being
7. Economic consequences (Economy). Messages about the economic consequences of the EHEC outbreak for private citizens, entrepreneurs, or the economy in general, and the actions that governments take in order to minimize these consequences

**Table 4:** Origin and content of messages consumed by Dutch participants (total number of codings = 129).

	Presence	Preventive	Deaths	Infections	Spread	Pathogenesis	Economy
TV	6	1	0	1	3	0	0
Radio	9	5	2	3	4	0	2
Newspaper	6	6	10	3	3	2	7
Internet	14	5	7	3	2	1	6
Total (% of total codings)	35 (27.13%)	18 (13.95%)	19 (14.73%)	10 (7.75%)	12 (9.30%)	4 (3.10%)	15 (11.63%)

**Table 5:** Origin and content of messages consumed by German participants (total number of codings = 235).

	Presence	Preventive	Deaths	Infections	Spread	Pathogenesis	Economy
TV	14	10	4	7	5	5	4
Radio	9	6	2	3	1	3	2
Newspaper	6	5	2	4	1	3	2
Internet	22	17	10	13	6	10	4
Total (% of total codings)	52 (22.13%)	40 (17.02%)	19 (8.09%)	27 (11.49%)	13 (5.53%)	22 (9.36%)	12 (5.11%)

In the Dutch context, most messages focused on the presence of the EHEC bacteria on a product, followed by messages about the number of EHEC-related deaths and preventive measures that were taken by the government or could be taken on an individual level. In Germany, more attention was focused on the preventive measures and the pathogenesis of the EHEC bacteria. Messages on the number of deaths, economic consequences of the outbreak, and its geographical spread were not broadcast as often.

### Conversations About EHEC

Our participants held 39 conversations; 13 of these conversations were reported by Dutch participants, 26 by German participants. Most conversations were held with friends (19), followed by conversations with family members and colleagues.

The conversations held by the Dutch participants covered a wide range of topics. However, one topic was discussed most: dietary choices. Several participants talked with other people about whether or not they should consume certain types of food.

NL5 With a friend I talked about eating “possibly infected food”. We were eating and also ate cucumber. Nonetheless, everybody dared to eat it, as we know that chances of infection are just very small.

Among the German participants, three topics were discussed most. First, they discussed dietary choices. Second and related, they talked about the products on which the EHEC bacteria were found. Third, they talked about the reliability of the media coverage of the outbreak and discussed their critique of it.

### Active Information Seeking

**Figure 3:**
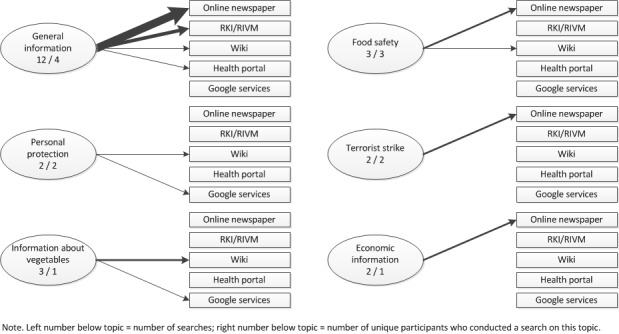
Searches for EHEC information: topic and information source.

In total, the participants reported 24 searches for EHEC-related information on their event-contingent forms: 7 by Dutch participants and 17 by German participants. All of these searches were conducted over the Internet, mostly with Google as a starting point. Figure 3 gives an overview of the information the participants sought and where they found it. We could discern 6 types of information that participants needed.

#### General Information

Several participants felt a need to search for general information on EHEC: “How can one be infected with EHEC?” (G1), or “What is the current situation on EHEC like?” (G8). Several German participants wanted to know more about the current situation on EHEC, after not having heard about it in the mass media for a few days. All but one search was concluded successfully. Most participants thought their question was very important and found an answer in an article published by an online newspaper. Some German participants also used the website of the RKI to get informed. All searches were concluded with a satisfactory or very satisfactory result.

#### Food Safety

Three participants wondered at some point in time whether a certain type of vegetable was infected with the EHEC bacteria. These searches differed in importance to the participants and were all concluded to a satisfactory level by looking up information on online newspapers or a wiki.

#### Personal Protection

Two persons had a question about personal protection, which they believed important. One person (NL3) had a general question: “How can I protect myself against EHEC?”, while the other person (NL8) wanted to know whether a town in which EHEC was diagnosed was nearby the residence of her parents.

#### Terrorist Strike

Two German participants wondered whether the EHEC outbreak was the result of a terrorist strike. This information was very important to them, and they both found an answer to their question in an online newspaper with which they were satisfied or neutrally satisfied.

#### Information About Vegetables

One participant (NL8) wanted more information about vegetables, like “I wanted more general information about bean sprouts because I don’t really know what it looks like.” To her this information was (very) important. She found her information on Wikipedia or Google images. She was either satisfied or neutrally satisfied with the information she found.

#### Economic Information

One participant (G1) sought information about the economic consequences of the EHEC outbreak. These matters were very important to her. She found a very satisfactory answer to her questions in online newspapers.

A topic that was brought forth by some participants on their diary entries was source credibility*.* Several participants indicated that they valued a trustworthy source for information on this particular topic. One participant said:

G6 I know the Robert Koch Institute, but there are many people who don’t know this serious source and rather use Bild [a German tabloid newspaper focused on sensational news] to get informed. I don’t think this newspaper is a suitable source to get informed about EHEC!

Apparently, not all participants valued source credibility that much. One participant (NL3) who searched for information on “How to protect myself against EHEC?” (see above) found information on a Dutch wiki-like website. The writer of this information was an amateur who had also written articles on the website with the titles “How to find a good restaurant” and “How to save energy with glass wool”. Nonetheless, participant NL3 was highly satisfied with the information provided by this amateur.

Finally, we expected there to be some interaction between passive information consumption and actively searching for information. For example, two friends discussing the safety of cucumbers could have led to a Google search about the vegetable’s safety. However, our data did not uncover such patterns. This suggests that during an infectious disease outbreak, active information searches are relatively small, personal activities.

### Satisfaction With Information Provision

The Dutch participants were reasonably satisfied about the information provision on the EHEC outbreak. They appreciated the high frequency of information updates and the honest manner in which information was communicated. However, they noted a lack of information from local or national government organizations but did not really mind as the risks associated with the outbreak were small in the Netherlands. The greatest source of dissatisfaction for the Dutch participants were the warnings on the safety of vegetables. Often, these were given for vegetables that turned out to be perfectly safe. This resulted in uncertainty about what to do.

NL5 I understand that they are eager to show to people that they are working hard at it, and that they have probably found a source, but I’d rather that they’d wait with strong statements until they are sure. Now producers of tomatoes and cucumbers have high damages because, in the beginning, they were suspected of being the source. If researchers don’t know, then they should just say so and should not cause confusion or panic by hastily drawing conclusions.

The Dutch participants were unanimous about information they lacked most: information about the conclusion of the outbreak. In their eyes, the media coverage of the outbreak silently came to an end and they asked for the media to inform the public of the end of the outbreak and a final verdict of its source. The preferred information source during the EHEC outbreak was the Internet for Dutch participants, and especially one news website (nu.nl).

For the German participants, being satisfied or not seemed to hinge on two issues: (1) was it made clear or not to the participant what the source of the outbreak was, and (2) did they find a single, credible source that provided all information in a well-written manner? Other sources of satisfaction were the warnings they received for different kinds of vegetables, while others appreciated the high frequency of information updates. In line with the Dutch participants, the German participants were also not happy with the constantly changing warnings about the safety of different types of food. They also disliked the alarming tone of the media and the different government organizations blaming each other for the long time it took to find the source of the outbreak. Among the German participants, the preferred sources were diverse: the television news, a German news channel (NTV), the radio, and finally, several websites like Yahoo! news, the RKI website, and the Spiegel newspaper website. Interestingly, several participants stated their preference to check EHEC-related news on mobile devices, using apps provided by news media.

### Use of Social Media

None of the Dutch participants used Twitter as an information source, either because they did not use Twitter (for receiving this kind of information), or they thought it was not a reliable source. Several German participants did use Twitter for receiving information, via the Twitter feed of online news sites, TV news channels, or the dedicated Twitter feed @EHEC_Watch. The German participants who did not use Twitter thought Twitter was not reliable enough or was not a suitable medium for this kind of news. The Dutch participants also did not use Facebook or Hyves to get informed about EHEC. They thought these social networks were meant for communicating with friends only and thought information provided here was unreliable. The German participants had similar reasons for not using Facebook or StudiVZ: they thought information on social networks was unreliable or wanted to use them only for communicating with friends.

## Discussion

Crisis communication literature suggests that Web 2.0 technologies can be valuable instruments for organizations for informing the public and keeping them involved [35], and the uprisings across the Middle East and North Africa have suggested the same. Our results indicate, however, that social media (like Facebook and Twitter) are not seen as suitable or reliable sources for communicating information during an infectious disease outbreak. These media are primarily viewed as a tool for communicating with friends. Health organizations presenting themselves on such forums to their public to inform them about an outbreak would be viewed “out of place”. And for many people, health-related information communicated via Facebook or Twitter would render it unreliable outright. Despite these strong negative feelings, several participants did use Twitter for keeping up to date with the latest news on the outbreak. The senders of these tweets, however, were primarily traditional media outlets like newspapers or a dedicated Twitter news feed from an expert source. This suggests that for a certain group of people, Twitter is a suitable source for being updated during an infectious disease outbreak, but only if the source’s credibility is spotless. One 2.0 service that was used more often were wikis. When people were actively searching for information, they used these collaborative efforts. Apparently, people view wikis as extensive and useful information sources, and source credibility does not play an important role here. We find this remarkable, as often these wikis are written by amateur writers who lack the necessary expertise. There may be work here for health organizations to keep an eye on, and if necessary, contribute to wikis during an infectious disease outbreak (eg, pages about the pathogen or disease carriers).

An item that we have already discussed but that affects more media than social media alone is source credibility. Most people attached great importance to receiving reliable information from a credible source. As other studies have already pointed out, quality seals can enhance the credibility of information [36] or can increase compliance with health advice [37]. We think that the use of these seals should be increased, but this presents problems for many websites that publish user-generated content, like wikis, as their content is constantly changing. The development of automated information quality evaluation tools [38], which use marker constructs such as the presence of an editorial review to determine information quality “on the fly” may solve this problem.

The diaries and pre- and postsurveys identified several citizen information needs during an infectious disease outbreak that health organizations should watch out for. They should:

1. Keep citizens updated on the status of the outbreak. Citizens want to know just how bad the situation is at a given moment and what caused the outbreak. For this, they primarily rely on media such as news websites, or traditional news outlets, such as newspapers (in print or online) and television news (either via television or their website). The primary function of health organizations here would be informing these media.
2. Help citizens in protecting themselves. Information on how to protect oneself (“How can I prevent being infected?” Or in the context of the EHEC outbreak, “Which types of food are safe for me to eat?”, “Can I safely travel to Germany?”) is, for the most part, broadcast by the same news media: news websites, newspapers (in print or online), and television news (either via television or their website). Additionally, they refer to friends, family, or colleagues, or they search for information on the Internet. In case of the latter, they not only refer to online newspapers but also to Web 2.0 services like wikis and maps. Health organizations need to inform the media about this topic and, where possible, monitor and contribute to relevant Web 2.0 services (eg, by contributing to relevant Wikipedia pages).
3. Communicate the end of the outbreak. In case of an infectious disease outbreak, citizens need closure. Health organizations need to relay a firm statement at the end of an outbreak to citizens, with a clear description of its cause. As this information is related to the current status of the outbreak, it makes sense to broadcast this information via the media that citizens use to stay up to date about the outbreak.
4. Provide unequivocal information. In Germany during the EHEC outbreak, citizens received different messages from the organizations and administrators, while these administrators also blamed each other for the slow progress in finding the cause of the outbreak. This caused dissatisfaction among the participants in our study. During an infectious disease outbreak, government organizations and administrators should unite and talk with one voice.

Finally, in our study we assessed the information behavior of people living at the epicenter of an infectious disease outbreak and people living further away. The media coverage of the outbreak was greater at the epicenter than in a region 300 kilometers away. The outbreak kept citizens in the epicenter busier than people at distance: they talked more about the outbreak with others and more often searched actively for information. The information needs also differed. Citizens at a distance took a more passive stance towards gathering information and used the same information sources. Citizens at the epicenter used a wider variety of information sources. The factors that led to (dis)satisfaction between the two different groups were quite similar. Health organizations can rely on the news media when they have to deal with an infectious disease outbreak where the epicenter is not in the immediate vicinity. When a health organization has to deal with an infectious disease outbreak in their service region, they need to monitor and provide information as described above.

### Limitations of the Study

When conducting a diary study, one ideally wants to collect data from the beginning of the outbreak to the end. In practice, this is impossible as one cannot predict when and where an infectious disease outbreak will take place. As a result, researchers can only contact organizations that will help them with recruiting participants when an infectious disease outbreak is deemed worthy of investigation. This made finding organizations that had the time and energy available extremely difficult. Local health authorities were the ideal partners, but they were very busy with the outbreak. After trying to collaborate with them for some time, we realized this would not work and resorted to an alternative set-up: collaborating with universities and using student participants.

This has led to two limitations of this study. First, we lost valuable time finding participants. This means that we could not collect data covering the pre-crisis and initial crisis phase, but only during the maintenance and resolution phase (classification by [4]). We therefore missed the opportunity to collect data about the participants’ first confrontation with the infectious disease. We tried to compensate for this by asking them about it in the demographics survey, but many participants were already unable to recall this experience. In the future, this shortcoming could be avoided by, for example, conducting interviews with people on the street right after an infectious disease outbreak has been announced. Second, we used student participants, who are not representative of the general population: they are young, healthy, and do not necessarily have responsibility for a family. Furthermore, a number of them studied health sciences, and as a result, they might have had a relatively high knowledge level about infectious diseases or a relatively high interest in the outbreak. Students are also not “average” 2.0 media users but are considered heavy users. It is therefore interesting that our results showed little use of social media. If our participant group did not use it significantly, the total population most probably would not either, or even to a lesser extent.

Finally, the scope of this study has been limited. We have investigated one outbreak with a small, selective group of respondents. However, this study is the first of its kind: it addresses information behavior from the citizens’ point of view in depth and is the first to critically investigate the role of Web 2.0 media in this context. Previous research has analyzed Web 2.0 media output only, assuming that these media are useful and widely used during an infectious disease outbreak. We are the first to have taken a closer look at the truth behind this assumption. The explorative, qualitative nature of our study limits its generalizability but provides valuable input for future quantitative research on this topic.

### Conclusions

In this study, we investigated the use of Web 2.0 media during an infectious disease outbreak. Contrary to the prevalent opinion, social media (like Facebook and Twitter) played only a marginal role in the information provision towards citizens. Wikis, however, did play a role. They were consulted when people actively searched for outbreak-related information. Future research should therefore focus on the role wikis can play for informing the public during an infectious disease outbreak.

The findings of this study should be verified for different kinds of outbreaks. The EHEC outbreak was large. Citizens’ information behavior may differ for infectious disease outbreaks on a smaller scale, like a scabies outbreak in a nursing home. In addition, health organizations often have different procedures for these kinds of relatively small outbreaks, which influences speed of information dissemination and media coverage.

One goal that 2.0 media can serve, but which we have not discussed in this paper, is informing journalists. This is a distinctly different population from citizens with their own information needs and behavior. It is possible that social networks can be valuable for health organizations when communicating with these professionals. When several health organizations are involved in the outbreak and each owns a part of the communication to stakeholders (as in the German context), journalists should be the people who make sense of press releases and communicate this to the public at large. Studies should assess whether this is actually the case and what form this communication should take.

## Multimedia Appendix 1

Interval-contingent diary form given to the participants to record their passive information behavior.

## Multimedia Appendix 2

Event-contingent diary form given to the participants to record their active information behavior.

## Multimedia Appendix 3

Final coding scheme for content of passively consumed EHEC-related messages.
