# Supplementary material for: Should Health Organizations Use Web 2.0 Media in Times of an Infectious Disease Crisis? An In-depth Qualitative Study of Citizens’ Information Behavior During an EHEC Outbreak
Source: J Med Internet Res. 2012 Dec 20;14(6):e181. doi: 10.2196/jmir.2123 (PMC3799610; doi:10.2196/jmir.2123)
Supplement: Supplementary file 1 [file jmir_v14i6e181_app1.pdf]

Multimedia Appendix 1: Interval-contingent diary form

Where did you see, hear or read something about the EHEC bacteria?		
1. TV		
	On<br>[date entry field]	I saw on [channel/program entry field]<br>that [message entry field]
2. Radio		
	On<br>[date entry field]	I heard on [station/program entry field]<br>that [message entry field]
3. Newspaper		
	On<br>[date entry field]	I read in the [newspaper entry field]<br>that [message entry field]
4. Internet		
	On<br>[date entry field]	I saw on [Internet site entry field]<br>that [message entry field]
5. Somewhere else		
	On<br>[date entry field]	I saw or read that [message entry field]<br>Where did you see, hear or read this? [source entry field]
6. Did you talk with anyone about the EHEC bacteria?<br>No<br>Yes<br>If yes, with whom and what did you talk about?<br>With a [dropdown field with possible conversation partners] I talked about [topic of discussion entry field]		
7. Do you have any additional remarks or questions?<br>[entry field]		

Note. Each part of the form (TV, radio, etc.) consisted of five entry fields.
