# Supplementary material for: Should Health Organizations Use Web 2.0 Media in Times of an Infectious Disease Crisis? An In-depth Qualitative Study of Citizens’ Information Behavior During an EHEC Outbreak
Source: J Med Internet Res. 2012 Dec 20;14(6):e181. doi: 10.2196/jmir.2123 (PMC3799610; doi:10.2196/jmir.2123)
Supplement: Supplementary file 2 [file jmir_v14i6e181_app2.pdf]

Multimedia Appendix 2: Event-contingent diary form

Date:	Time:			
1. What question did you have about the EHEC bacteria? Or where did you want to know more about?				
2. How important was this for you?				
Very unimportant	Unimportant	Neutral	Important	Very important
3. Where did you search for an answer or for more information?				
4. Did you find an answer or the information you were looking for?<br>No, go to question no 7.<br>Yes, go to question no 5.				
5. Where did you find your answer or the information you were looking for?				
6. How satisfied were you with this answer or this information?				
Very unsatisfied	Unsatisfied	Neutral	Satisfied	Very satisfied
7. Do you have additional remarks or questions?				
