# Supplementary material for: Should Health Organizations Use Web 2.0 Media in Times of an Infectious Disease Crisis? An In-depth Qualitative Study of Citizens’ Information Behavior During an EHEC Outbreak
Source: J Med Internet Res. 2012 Dec 20;14(6):e181. doi: 10.2196/jmir.2123 (PMC3799610; doi:10.2196/jmir.2123)
Supplement: Supplementary file 3 [file jmir_v14i6e181_app3.pdf]

Multimedia appendix 3: Final coding scheme for content of passively consumed EHEC-related messages

Code	Definition	Example
**Presence EHEC bacteria**	Messages about the (proven or not proven) prevalence of the EHEC bacteria in a country, on a product(group), on a company or in a river, or messages about the starting point of the EHEC outbreak	"That Egyptian sowing seeds may be behind the EHEC bacteria"
**Number of deaths**	Messages about the number of deaths as a result of the EHEC outbreak	"At this moments 37 people have died due to the consequences of EHEC."
**Economic consequences**	Messages about the economic consequences of the EHEC outbreak for private citizens, entrepreneurs, or the economy in general, and the actions that governments take in order to minimize these consequences	"The tax services help duped vegetable producers by lowering the provisional tax assessment, postponing the payment or by arranging an alternative payment arrangement. Producers should turn to the tax service quickly."
**Geographical spread**	Messages about a specific area where the EHEC bacteria has been encountered or could be present for the first time, where people have become ill for the first time, or have died for the first time as a result of the EHEC bacteria	"Sprouts in England have been infected as well."
**Number of infections**	Messages about the number of infections as a result of the EHEC outbreak	"The number of infections with the EHEC bacteria has risen to 11"
**Knowledge about vegetables**	Message about the knowledge people have of different kinds of vegetables	"People did not know what a red beet's sprout was"
**Pathogenesis of EHEC**	Messages about the way the EHEC bacteria spreads or infects a human being	"EHEC can also be spread from humans to food"
**Control / treatment of EHEC-related diseases**	Messages about the way an EHEC infection is treated (medical) or an EHEC infection is diagnosed (in a human being or on a product (group))	"Researchers of the Radboud University will try to bring a chip on the market, with which you can diagnose an EHEC infection on vegetables and fruit within half an hour."
**Preventive measures**	Messages about preventive measures that are being taken to prevent the spread of, or infection with the EHEC bacteria, including messages about the (un)safety of a specific product group	"The EU have now declared an import ban for sprouts and have destroyed all stock from before 2009."
**Functioning of politics / health care**	Messages about the functioning of politics and/or health care during the EHEC outbreak.	"The employees of hospitals are suffering high pressure because of the EHEC outbreak. A lot of them are down because of their powerlessness in the current situation."
**Individual EHEC stories**	Messages about personal histories during the EHEC outbreak	"A Dutch physician works in a hospital in Bremen. He describes his work as a physician and how he deals with the EHEC bacteria."

**Pointers to more information**	Messages about sources where information about the EHEC bacteria can be found	"The health insurer for technical personnel has opened a hotline, for people with questions about EHEC."
**Public behavior as a result of the EHEC outbreak**	Messages about public actions or behavior as a result of the EHEC outbreak	"A lot of people still avoid eating salad."
**Legal actions**	Messages about legal actions, taken as a result of the EHEC outbreak	"The public prosecutor will start a criminal investigation against the Biohof in Biennebüttel (Niedersachsen)."
**Future**	Messages about future actions and expectations	"In the future, producers and importers of sprouts will be more strictly controlled."
**Medical consequences**	Messages about the medical consequences people with an EHEC infection will have to deal with	"EHEC patients that are fired from the hospital will have to undergo treatment for another year."
